# Clinicopathological Factors Predisposing to No. 12a Lymph Node Metastasis in Gastric Cancer: A Prospective Cohort Analysis

**DOI:** 10.1002/cnr2.70239

**Published:** 2025-06-11

**Authors:** Amirmohsen Jalaeefar, Habibollah Mahmoodzadeh, Mohammad Shirkhoda, Ramesh Omranipour, Seyed Rouhollah Miri, Narjes Mohammadzadeh, Arshia Zardoui, Amirsina Sharifi

**Affiliations:** ^1^ Department of Surgery, Subdivision of Surgical Oncology Cancer Institute, Tehran University of Medical Sciences Tehran Iran; ^2^ Department of Surgery Imam Khomeini Hospital Complex, Tehran University of Medical Sciences Tehran Iran; ^3^ Sina Trauma and Surgery Research Center Tehran University of Medical Sciences Tehran Iran

**Keywords:** gastric cancer, lymph node excision, lymph node metastasis

## Abstract

**Background:**

The current standard surgical procedure for gastric cancer (GC) is gastrectomy and D2 lymphadenectomy, which includes harvesting No. 12a lymph node (LN) station.

**Aim:**

The purpose of this study was to identify the clinicopathologic factors associated with No. 12a lymph node metastasis.

**Methods and Results:**

Eighty‐nine patients with GC undergoing gastrectomy and D2 lymphadenectomy were included in this single‐arm prospective cohort study. Logistic regression analyses were used to clarify the correlation between No. 12a involvement and clinicopathologic characteristics. Eighty‐nine patients (66% males) with a mean age of 58.86 ± 13.06 years were included. The upper third of the stomach was the most common tumor site (43.8%). neoadjuvant chemotherapy (NAC) was administered to 77 patients (86.5%). Total gastrectomy was the most common surgical procedure (67.4%), and 49.4% of tumors were poorly differentiated. Ten patients (11.24%) had 12a LN metastasis. Patients with 12a LN involvement exhibited greater number of harvested LNs in other stations (28.5[27–39.25] vs. 25[21–30], *p =* 0.024) and a higher presence of LN involvement in other stations (22[11–32] vs. 0[0–4], *p* = < 0.001). Univariate logistic regression analysis showed that the number of harvested other nodes (OR: 1.11[1.02–1.21]), number of involved other nodes (1.23[1.11–1.37]), omental involvement (OR: 10.86[1.84–64.24.57]), lymphovascular invasion (6.90[1.37–34.70]), and perineuronal invasion (OR: 6.16[1.23–31.11]) were significantly associated with No. 12a station metastasis. However, in multivariate logistic regression, only the number of involved other nodes showed a significant association with No. 12a station metastasis (OR: 1.30[1.09–1.55]). There was no difference between patients who received NAC and who did not in terms of No. 12a involvement (*p* value = 0.61).

**Conclusion:**

Among clinicopathologic risk factors, involvement of other lymph node stations was significantly associated with No. 12a lymph node metastasis. Therefore, No. 12a lymph node dissection should be considered in patients with advanced gastric cancer.

## 1 Introduction

Gastric cancer (GC) presents a significant global health challenge, being ranked as the fifth most widespread and the fourth principal cause of cancer‐related mortality worldwide [1]. Surgical resection combined with lymphadenectomy remains pivotal in the management of GC, particularly in cases of locally advanced disease [2, 3]. Nevertheless, there are persistent debates concerning the scope of lymph node (LN) dissection and its impact on patient outcomes [4]. Specifically, there is ongoing debate over the necessity of dissection of the hepatoduodenal ligament LNs (No. 12a) in D2 gastrectomy (gastrectomy and D2 lymphadenectomy), with conflicting opinions on its influence on long‐term survival.

Studies highlight the importance of LN dissection in accurately staging GC and ultimately improving prognosis [5]. The adoption of D2 lymphadenectomy (removal of lymph nodes along the right and left cardiac, lesser and greater curvature, suprapyloric along the right gastric artery, and infrapyloric area. In addition to those along the left gastric artery, common hepatic artery, celiac artery, and splenic artery) proposed by the Japanese Gastric Cancer Association has gained widespread acceptance as the gold standard for advanced GC management [5, 6]. Nevertheless, the inclusion of No. 12a LNs in this protocol has been met with varying levels of compliance in actual practice, with some studies reporting notably low adherence rates [2, 7]. This noncompliance prompts an essential question: does neglecting No. 12a LN dissection compromise long‐term survival, and which patients stand to benefit most from this procedure?

Additionally, research highlights that LN metastasis remains a critical factor influencing outcomes in GC patients, thereby impacting treatment strategies and prognostic considerations [8]. The location and depth of tumor invasion significantly influence the rate of LN metastasis, emphasizing the need for tailored approaches in gastrectomy and LN dissection [3]. Recent updates in guidelines for GC management emphasize the importance of a multidisciplinary approach, with gastrectomy and thorough LN dissection as central components [4, 9]. The purpose of this study was to evaluate the clinicopathological factors associated with No. 12a lymph node metastasis in gastric cancer and to assess whether routine dissection of the No. 12a lymph node remains necessary in the context of neoadjuvant chemotherapy. The present study seeks to provide insights into the distribution of LN metastasis in the 12a station of GC, with the ultimate goal of refining surgical strategies for determining the extent of lymphadenectomy in this specific patient cohort.

## 2 Methods

### 2.1 Study Design

This study was designed as a single‐arm prospective cohort involving GC patients who met predefined criteria, and they were subsequently monitored to assess long‐term survival outcomes. This article outlines the patient enrollment process, and the second part will be discussed after a minimum follow‐up period of 3 years. The initial phase was carried out on a consecutive cohort of GC patients who underwent curative surgery at the Cancer Institute, Tehran University of Medical Sciences (TUMS), Tehran, Iran, from March 2022 to August 2023. Following approval from the institutional review board and ethics committee of TUMS (IR.TUMS.IKHC.REC.1401.419), patients with histologically confirmed gastric adenocarcinoma were enrolled in the study. All enrolled patients underwent gastrectomy with R0 resection, the extent of which was determined by tumor location, coupled with lymphadenectomy in accordance with the National Comprehensive Cancer Network Guidelines [9]. Exclusion criteria encompassed patients with GC in the stomach remnant and those with metastatic tumors who exclusively received exploration or palliative surgery. Moreover, patients who did not undergo No. 12a LN dissection (undergoing D1/D1+ lymphadenectomy) were not considered in this study. In all cases regardless of gastric resection extent, No. 12a LNs were meticulously dissected, isolated from the specimen immediately during the operation, and subsequently forwarded separately for pathological evaluation. Concurrently, other dissected LN stations were also subject to evaluation.

It should be mentioned that operating chief surgeons involved in the study had more than 10 years of experience in the D2 gastrectomy and the extent of lymph node dissections was the same.

Station 12a LNs, located along the proper hepatic artery within the hepatoduodenal ligament, were categorized based on the classification provided by the JGCA (3rd English edition) [6]. The anatomic boundaries of No. 12a LNs were specified as follows: upper border: the point of convergence of the left and right hepatic arteries; lower border: the origin of the proper hepatic artery at the upper boundary of the pancreas; lateral border: the left margin of the common bile duct; left border: the left edge of the hepatoduodenal ligament; anterior border: the front aspect of the hepatoduodenal ligament; posterior border: the front wall of the portal vein.

Patient allocation for neoadjuvant chemotherapy was based on NCCN guideline [9]. The chemotherapy regimen was Fluorouracil, leucovorin, oxaliplatin, and docetaxel (FLOT) and it was administered every 2 weeks (q2w) for 4 cycles preoperatively and 4 cycles postoperatively:

- Fluorouracil (5‐FU, F): 2600 mg/m^2^ IV as a 24‐h continuous infusion on Day 1.
- Leucovorin (Folinic Acid, L): 200 mg/m^2^ IV on Day 1.
- Oxaliplatin (O): 85 mg/m^2^ IV on Day 1.
- Docetaxel (T): 50 mg/m^2^ IV on Day 1.

In the subsequent phase of the study, all patients will undergo periodic follow‐up through outpatient visits. The follow‐up schedule will entail assessments every 3 to 6 months within the first 2 years following surgery, every 6 to 12 months in the subsequent 3 years, and then annually thereafter. In this study, the primary endpoint of interest is overall survival (OS), which will be calculated from the date of surgery to the date of death from any cause or to the date of the most recent follow‐up.

### 2.2 Data Collection and Variables

From the medical records, the following variables were documented: demographic variables (age and sex), tumor location, duration from chemotherapy/chemoradiotherapy to surgery, extent of gastrectomy, histology type, presence of perineural invasion, presence of lymphovascular invasion, and preoperative complete blood count variables. Patients were categorized into four groups based on tumor location: the upper third (including cardia and fundus), middle third (body), lower third (including antrum and pyloric area), and entire (linitis plastica) of the stomach. The numbers of harvested LNs and metastatic LNs were examined based on postoperative pathologies. Pathological staging was conducted in accordance with the 8th edition of the American Joint Committee on Cancer (AJCC) stage system for GC. Our outcome variable was the involvement of the 12a lymph nodes divided into two categories: yes or no.

### 2.3 Sample Size Calculation

The sample size was calculated using G*Power 3.1 for logistic regression. The primary outcome was 12a lymph node positivity, with the number of harvested LNs as the key predictor. Based on prior studies, the baseline proportion of 12a positivity was set at 5% (0.05) [2], and the odds ratio for harvested LNs was assumed to be 1.5. Using a two‐tailed test, *α* = 0.05, power = 0.80, and R^2^ = 0.1 to account for other predictors, the required sample size was determined to be 87 patients. Our final sample of 89 patients exceeded this requirement. Post hoc power analysis was performed with G*Power version 3.1 [10].

### 2.4 Statistical Analyses

Categorical variables were presented as frequencies and percentages, while quantitative variables were expressed as means with standard deviation, and median with interquartile range. In some cases, there were missing values, with the highest proportion being 6%. These missing data were managed using the pairwise deletion method [11]. Prior to analysis, data were assessed for normal distribution using the Shapiro–Wilk test. For the comparison of categorical variables, chi‐square and Fisher exact tests were employed. Quantitative variables were compared using either the *T*‐test or the Mann–Whitney test, depending on the normality of the data. Linear regression was used to find out collinearity. To identify potential factors associated with station 12a involvement, both univariate and multivariate regression analyses were conducted. The findings were reported as odds ratio (OR) and 95% confidence interval (CI). All statistical analyses were carried out using IBM SPSS version 22.0, SPSS Inc., Chicago, IL. Statistical significance was defined as *p*‐values < 0.05.

## 3 Results

### 3.1 Patients' Clinical and Pathological Characteristics

**FIGURE 1:**
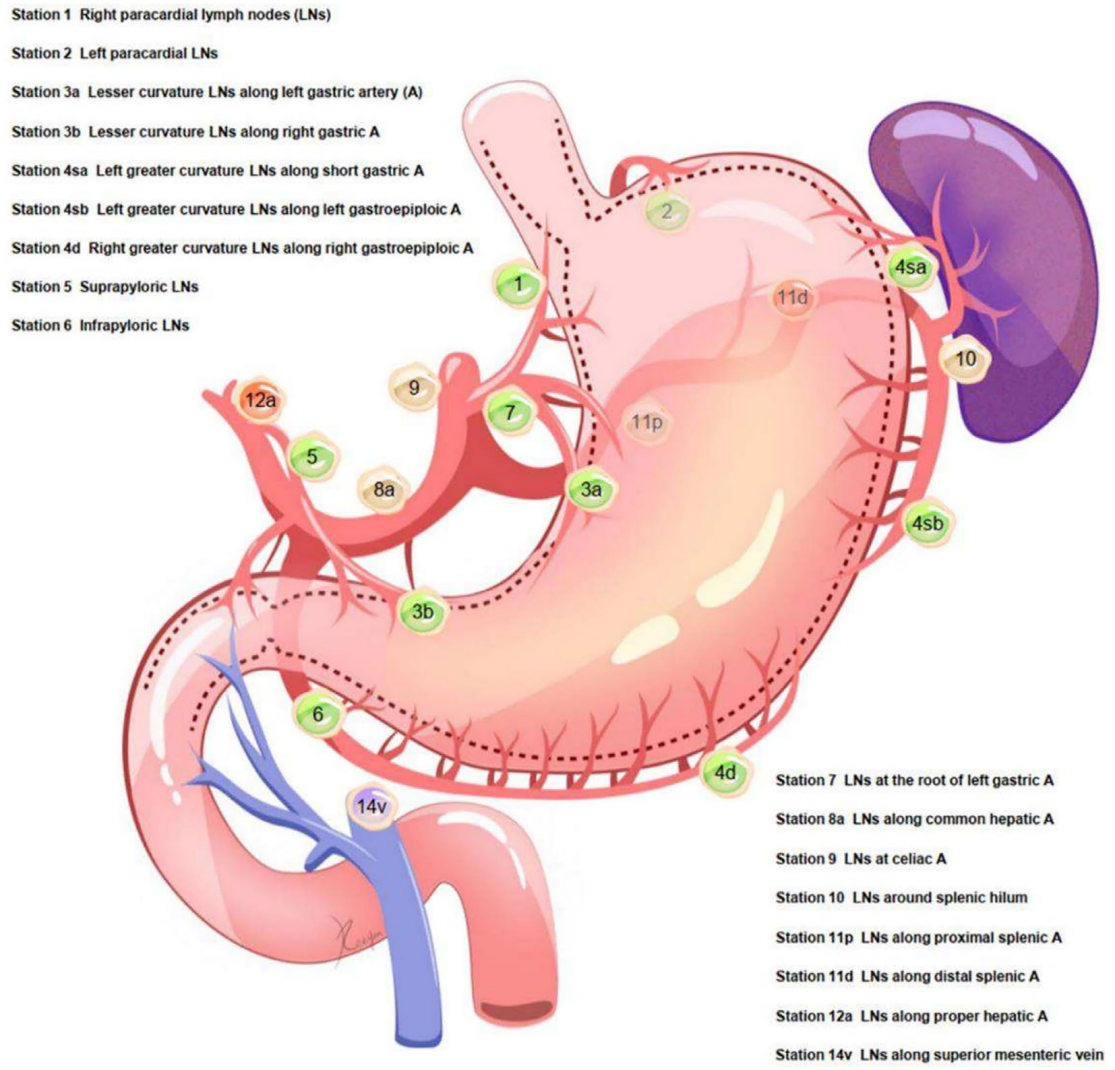
Details of Ivmph node stations [24].

Eighty‐nine patients with GC, with a mean age of 58.7 ± 13.1 years old, were eligible to be included in this study. Of these 89 patients, 59 (66.3%) patients were males. The upper third of the stomach was the most common site of the primary tumor in these patients (*n* = 39; 43.8%). Prior to surgery, 77 patients (86.5%) underwent neoadjuvant chemotherapy (NAC) and 5 patients (5.7%) underwent neoadjuvant chemo‐radiotherapy. The compliance rate for neoadjuvant chemotherapy was 100% and all patients who were assigned for neoadjuvant treatment received it. The mean number of days between the last chemotherapy or chemo‐radiotherapy session and surgery was 30.24 ± 18.36 days. Total gastrectomy was the most common surgery in this study in 60 (67.4%) patients. All patients underwent 12a and other stations LND. Table 1 and Figure 1 present the patients' characteristics in detail.

**TABLE 1:** Patients' characteristics.

Variables	Total *N* = 89	12a not Involved *N* = 79	12a Involved *N* = 10	*P*
Age (years)	58.9 ± 13.1	58.0 ± 13.3	65.9 ± 9.0	0.070
Gender				0.485
Male	59 (66.3)	51 (64.6)	8 (80.0)	
Female	30 (33.7)	28 (35.4)	2 (20.0)	
cT				**0.030**
T1	3 (3.4)	3 (3.8)	0 (0)	
T2	13 (14.6)	13 (16.5)	0 (0)	
T3	42 (47.2)	40 (50.6)	2 (20.0)	
T4	31 (34.8)	23 (29.1)	8 (80.0)	
cN				0.308
N0	19 (21.6)	19 (24.4)	0 (0)	
N1	17 (19.3)	14 (17.9)	3 (30.0)	
N2	31 (35.2)	27 (34.6)	4 (40.0)	
N3	21 (23.9)	18 (23.1)	3 (30.0)	
Tumor location				0.353
Upper third	39 (43.8)	36 (45.6)	3 (30.0)	
Middle Third	17 (19.1)	16 (20.3)	1 (10.0)	
Lower Third	29 (32.6)	24 (30.4)	5 (50.0)	
Linitis plastica	4 (4.5)	3 (3.8)	1 (10.0)	
NAC				0.619
Yes	77 (86.5)	69 (87.3)	8 (80.0)	
No	12 (13.5)	10 (12.7)	2 (20.0)	
NACR				0.461
Yes	5 (5.7)	4 (5.1)	1 (10.0)	
No	77 (94.3)	74 (94.9)	9 (90.0)	
NAC sessions	4[4–5]	4[4–5]	4[1.5–6.5]	0.804
NACR sessions	0[0]	0[0]	0[0]	0.523
CSI, days	30[20–40]	29[19.5–40]	30[17.25–41]	0.714
Pre‐op WBC count/μL	6300[4950–7600]	6300[4800–7400]	6800[5350–8300]	0.461
Pre‐op LYM, %	29.5[23–37]	31[23–38]	24[22.5–31‐5]	0.063
Pre‐op NEUT, %	62[56.25–66‐75]	61[55–66]	68[62–71]	0.070
Pre‐op PLT count, x10^3^/μL	203[162–257]	195[162–255]	240[229.5–317]	**0.027**
NLR	2.12[1.55–2.86]	2.04[1.45–2.75]	2.56[2.07–3.04]	0.120
PLR	118.09[83.90–153.94]	115.59[79.60–153.20]	137.43[100.21–219.60]	0.113
Surgery Type				0.329
Total gastrectomy	60 (69.8)	55 (72.4)	5 (37.5)	
Subtotal gastrectomy	24 (27.9)	19 (25.0)	5 (37.5)	
Proximal gastrectomy	1 (1.2)	1 (1.3)	0 (0)	
Esophagogastrostomy	1 (1.2)	1 (1.3)	0 (0)	
Harvested 12a nodes	3[2–4]	3[1–4]	3[2–4]	0.389
Harvested other nodes	26[21–30]	25[21–30]	28.5[27–39.25]	**0.024**
Involved other nodes	1[0–7]	0[0–4]	22[11–32]	< **0.001**
Differentiation				0.391
Well	44 (49.9)	41 (51.9)	3 (30)	
Moderate	1 (1.1)	1 (1.3)	0 (0)	
Poor	34 (49.4)	37 (46.8)	7 (70)	
Omental involvement				**0.017**
Yes	6 (6.7)	3 (3.8)	3 (30.0)	
No	83 (93.3)	76 (96.2)	7 (70.0)	
LVI				**0.015**
Yes	37 (41.6)	29 (36.7)	8 (80.0)	
No	52 (58.4)	50 (63.3)	2 (20.0)	
PNI				**0.019**
Yes	39 (43.8)	31 (39.2)	8 (80.0)	
No	50 (56.2)	48 (60.8)	2 (20.0)	

*Note:* Quantitative data are presented as mean ± standard deviation and median [interquartile range]; Categorical variables are presented as numbers (percentages). Bold *p* values are statistically significant.

Abbreviations: cN: clinical N (staging); CSI: chemotherapy to surgery interval; cT: clinical T (staging); LVI: Lymphovascular invasion; LYM: lymphocyte; NAC: neoadjuvant chemotherapy; NACR: neoadjuvant chemoradiotherapy; NEUT: neutrophil; NLR: neutrophil to lymphocyte ratio; PLR: platelets to lymphocyte ratio; PLT: platelet; PNI: Perineuronal invasion; pre‐op: pre‐operation; WBC: white blood cell count.

### 3.2 12a Station LN Involvement

Post‐operation pathological results revealed that only 10 patients (11.24%) had 12a LN metastasis. Patients with metastasis of the 12a LN exhibited higher platelet counts (240[229.5–317] vs. 195[162–255], *p* = 0.027) and a greater number of harvested LNs in other stations (28.5[27–39.2] vs. 25[21–30], *p* = 0.024). Furthermore, these patients demonstrated a higher presence of LN involvement in other stations (22[11–32] vs. 0[0–4], *p* = < 0.001). Patients with No. 12a station involvement had a higher possibility of being in pT4 stage (*p* = 0.030) in a way that 8 out of 10 patients with No. 12a station metastasis had a pT stage of 4. Moreover, No. 12a LN involvement was more likely to be present in patients with omental involvement, lymphovascular invasion, and perineuronal invasion (*p* = 0.017, *p* = 0.015, and *p* = 0.019, respectively, Table 1). There was no difference between patients who received NAC and those who did not in terms of No. 12a involvement (*p* value = 0.61).

To find out the variables associated with No. 12a station involvement, we conducted logistic regression analyses. Univariate logistic regression analysis showed that platelet count (OR: 1.01[1.00–1.02]), number of harvested other nodes (OR: 1.11[1.02–1.21]), number of involved other nodes (1.23[1.11–1.37]), omental involvement (OR: 10.86[1.84–64.24.57]), lymphovascular invasion (6.90[1.37–34.70]), and perineuronal invasion (OR: 6.16[1.23–31.11]) were significantly associated with No. 12a station metastasis. Collinearity testing with linear regression was performed to find out the possible collinearity between variables associated with No. 12a station involvement. The tests revealed no collinearity as the Variance Inflation Factor and the Tolerance indices were less than 5 and more than 0.1, respectively. In multivariate logistic regression after including the mentioned variables in the analysis, only the number of involved other nodes showed a significant association with No. 12a station metastasis (OR: 1.30[1.09–1.55]). (Table 2).

**TABLE 2:** Regression analyses of the clinicopathological factors associated with 12a metastasis.

Variables	Univariate analysis			Multivariate analysis^1^		
	OR	95% CI	*P*	OR	95% CI	*P*
Age	1.06	0.99–1.12	0.077			
Gender, Ref: female	2.20	0.44–11.06	0.340			
cT†			0.066			
T2	0	—	0.999			
T3	0.11	0.03–0.74	0.028			
T4		Reference				
cN			0.978			
N0	0	—	0.998			
N1	1.29	0.22–7.37	0.778			
N2	0.89	0.18–4.50	0.886			
N3		Reference				
Tumor location			0.462			
Upper third		Reference				
Middle Third	0.81	0.72–7.78	0.750			
Lower Third	2.50	0.55–11.45	0.238			
Linitis plastica	4.00	0.31–51.30	0.287			
NAC	0.58	0.11–3.13	0.526			
NACR	2.06	0.21–20.46	0.539			
NAC sessions	0.97	0.72–1.29	0.812			
NACR sessions	1.02	0.95–1.11	0.556			
SCI	1.00	0.97–1.04	0.831			
Pre‐op WBC count	1	1–1	0.986			
Pre‐op LYM, %	0.97	0.90–1.03	0.249			
Pre‐op NEUT, %	1.04	0.98–1.11	0.209			
Pre‐op PLT count	1.01	1.00–1.02	**0.028**	1.03	0.99–1.02	0.639
NLR	0.97	0.71–1.34	0.868			
PLR	1.00	1.00–10.1	0.121			
Surgery Type‡						
Total		Reference				
Subtotal	2.90	0.75–11.11	0.121			
Harvested 12a nodes	1.02	0.79–1.32	0.885			
Harvested other nodes	1.11	1.02–1.21	**0.022**	0.90	0.74–1.10	0.301
Involved other nodes	1.23	1.11–1.37	**< 0.001**	1.30	1.09–1.55	**0.004**
Differentiation§						
Well		Reference				
Poor	2.59	0.62–10.73	0.191			
Marginal involvement	1.33	0.14–12.37	0.800			
Omental involvement	10.86	1.84–64.24	**0.009**	3.11	0.01–1104.04	0.705
LVI	6.90	1.37–34.70	**0.019**	0.39	0–34.96	0.681
PNI	6.19	1.23–31.11	**0.027**	0.80	0.01–61.42	0.921

*Note:* Bold *p* values are statistically significant.

Abbreviations: cN: clinical N (staging); CSI: chemotherapy to surgery interval; cT: clinical T (staging); LVI: Lymphovascular invasion; LYM: lymphocyte; NAC: neoadjuvant chemotherapy; NACR: neoadjuvant chemoradiotherapy; NEUT: neutrophil; NLR: neutrophil to lymphocyte ratio; PLR: platelets to lymphocyte ratio; PLT: platelet; PNI: Perineuronal invasion; pre‐op: pre‐operation; WBC: white blood cell.

^†^ Stage T1 was not included in regression analysis due to its small size (*n* = 2).

^‡^ Surgery types of proximal gastrectomy and esophagogastrostomy were not included in regression analysis due to their small size (*n* = 1).

^§^ Tumor pathology type of moderate differentiation was not included in regression analysis due to its small size (*n* = 1).

^1^ Six variables with a *p*‐value of < 0.05 in the univariate logistic regression analysis were included in the multivariate logistic regression.

## 4 Discussion

Our findings indicate that the total number of involved other lymph nodes is significantly associated with station 12a lymph node involvement (OR 1.30, 95% CI 1.09–1.55, *p* = 0.004). While systemic factors, such as preoperative platelet count and local tumor‐specific factors like lymphovascular, invasion and perineural invasion were significant in univariate analysis, their effects diminished in the multivariable model. Moreover, certain local tumor factors, including tumor location and clinical *T* stage, were not significantly associated with 12a positivity in either univariate or multivariate analysis.

These results suggest that station 12a positivity is primarily reflective of the overall burden of nodal metastasis rather than specific systemic or local tumor characteristics. This highlights the importance of accounting for the total extent of lymph node involvement when predicting station 12a positivity and developing targeted treatment strategies.

LN metastasis emerges as a crucial prognostic factor for GC patients [12, 13]. The persistent debate regarding the inclusion of station No. 12a lymph node dissection in D2 lymphadenectomy has been a longstanding issue. In earlier classifications, positivity of station No. 12a LNs was regarded as a distant metastasis, whereas the 8th Cancer Staging Manual of the American Joint Committee on Cancer (AJCC) redefined it as a regional metastasis, justifying its inclusion in D2 lymphadenectomy alongside distal or total gastrectomy [14, 15]. Studies conducted by Shirong et al. [16] and Lee et al. [17] highlight that patients with station No. 12a LN metastasis exhibit markedly reduced 5‐year survival rates, accentuating the potential advantage of station No. 12a LN dissection for this specific patient subgroup.

The incidence of station 12a LN metastasis displays significant variability, ranging from 1.7% to 54.4% across different studies [18]. This variability arises from the diverse clinicopathological features of the patients recruited in each study and the adoption of different guidelines for managing gastric cancer, which may or may not involve neoadjuvant treatment. Hence, it is inevitable to obtain differing results. Additionally, distinguishing No. 12a LNs from those along the right gastric artery (No. 5) and the common hepatic artery (No. 8) post‐dissection proves challenging. These LNs are more susceptible to metastasis in GC patients. If there is a misinterpretation during pathological evaluation and these LNs are mistaken for No. 12a LNs, it could lead to an elevated positive ratio of No. 12a LN metastasis. In our study, we took measures to isolate No. 12a LNs immediately upon their dissection during the operation to prevent any potential confusion with other LNs.

Several factors have been reported as potential predictors of No. 12a LN metastasis, including tumor location, size, stage, soft tissue invasion, nerve invasion, intravascular cancer emboli, macroscopic type, and histological type [3]. In a study involving 2788 GC patients, Zhu et al. [5] identified older age, longitudinal tumor location (middle or lower third involvement), cross‐sectional tumor location (lesser curvature involvement), undifferentiated type, venous invasion, presence of cancer nodules, pT4 stage, and distant metastasis as independent predictors of station 12a LN metastasis. Notably, Zhu et al.'s study excluded patients who underwent NAC, and they observed that patients with No. 12a LN metastasis (excluding stage IV cases) exhibited significantly better overall survival compared to TNM stage IV patients. Our study did not confirm these associations, which may be due to differences in patient selection, sample size, or methodology. Unlike Zhu et al., who excluded patients with distant metastasis and neoadjuvant chemotherapy (NAC), our cohort, included a broader range of cases, which might have influenced the observed patterns of metastasis. Additionally, some discrepancies in reported incidence rates of station 12a LN metastasis across studies may stem from variations in LN dissection techniques, pathological assessment, and classification of LN stations.

In a study by Shu et al. [4], medical records of 413 consecutive GC patients who underwent radical gastrectomy with standard D2 lymphadenectomy, following the Japanese Gastric Cancer Treatment Guidelines, were investigated. The data from this study suggested that No. 12a involvement did not emerge as an independent prognostic factor. However, patients with No. 12a involvement displayed a poorer prognosis than those without. The overall incidence of No. 12a LN metastasis was low, but notably higher in patients with very advanced tumors (stage III/IV) involving the lower third of the stomach. The authors concluded that considering No. 12a LN dissection for these patients might lead to improved survival outcomes. Our cohort included patients with NAC, whereas Shu et al. primarily focused on patients undergoing primary surgery without prior systemic therapy. NAC can significantly alter the natural pattern of lymphatic metastasis, potentially masking tumor‐related predictors such as T stage and LVI.

In the assessment of lymphadenectomy quality, the total number of retrieved LNs is a widely accepted method, alongside compliance, though the latter has limitations as an indirect marker. The total number of retrieved LNs has been demonstrated to correlate with patient survival, and it is recommended to retrieve at least 15 LNs for accurate assessment of pathologic staging [19]. Additionally, in advanced gastric cancer, it is suggested that a total of 29 LNs should be retrieved to maximize the survival advantage of D2 lymphadenectomy [20]. However, LN dissection is a nuanced procedure that necessitates considerations of clinical tumor classification and location in order to determine the extent of lymphadenectomy. The number of resident LNs and appropriate compliance rate could vary based on individual and tumor characteristics. Therefore, there remains an unmet need for a comprehensive quality control assessment of lymphadenectomy, one that goes beyond relying solely on the total number of retrieved LNs, to enhance survival outcomes in gastric cancer [21].

In a study by Seo et al. [6], they investigated the impact on survival at individual LN stations in a cohort of 2932 patients who underwent radical gastrectomy with either D1+ or D2 LN dissection in accordance with the Japanese gastric cancer treatment guidelines. Patients who underwent neoadjuvant chemotherapy were excluded from the analysis. Their findings revealed that the highest compliance among extra‐perigastric stations was observed for station No. 8a (86.6%), followed by station No. 7 (76.6%) and station No. 9 (68.3%). In contrast, stations No. 11 and No. 12a exhibited low compliance rates of 28.9% and 31.0%, respectively. Compliance at stations No. 7, 8a, and 9 was associated with superior 5‐year relapse‐free survival rates compared to noncompliance. However, at stations No. 11 and 12a, there were no significant differences in relapse‐free survival between the compliance and noncompliance groups. In multivariable analysis, stations No. 7 and 8 alone exhibited an elevated hazard ratio for relapse‐free survival in the noncompliance group relative to the compliance group.

In a study conducted by de Jongh et al. [22], data from the LOGICA trial was utilized to analyze the pattern of metastases per LN (LN) station and its correlation with tumor location, cT‐stage, Lauren classification, and NAC. Among the 212 patients who underwent D2‐gastrectomy, 158 (75%) received NAC. LN metastases were observed in 120 patients (57%). Remarkably, despite NAC, each LN station (no. 1–9, 11 and 12a) showed metastases, irrespective of tumor location, cT‐stage, and histological subtype. While LN metastases were more prevalent in diffuse compared to intestinal tumors, this discrepancy was not observed for cT3–4 versus cT1–2 stage. However, the pattern of LN metastases exhibited similarity across these subgroups. This study argues against reducing the extent of lymphadenectomy after NAC for GC, emphasizing the importance of D2‐lymphadenectomy in Western patients. Compared to our findings, de Jongh et al. also suggested that the extent of overall LN involvement correlates more strongly with station 12a metastasis, which aligns with our results.

Furthermore, it should be emphasized that neoadjuvant chemotherapy might affect No. 12a involvement. Based on current literature, there is controversy between Eastern and Western guidelines regarding neoadjuvant chemotherapy [6, 9]. The approach to NAC underscores the disparity between these guidelines. While both perspectives acknowledge its potential in enhancing curative resection rates, the Eastern guidelines, particularly those from the Japanese Gastric Cancer Association (JGCA) [6], express reservations regarding its widespread application. The Korean Gastric Cancer Association (KGCA) [23] suggests that NAC, as part of perioperative chemotherapy, can be considered for patients with respectable locally advanced gastric cancer. This reluctance may be attributed to differences in trial data, patient demographics, and surgical practices across regions. In contrast, Western guidelines, such as those from the National Comprehensive Cancer Network (NCCN) [9], endorse NAC with specific regimens like FLOT or fluoropyrimidine‐based combinations. This discrepancy reflects varying levels of confidence and adoption in neoadjuvant approaches, potentially influenced by differences in disease presentation and patient characteristics. Therefore, the differing perspectives on the role of No. 12a dissection in patient outcomes could stem from distinct preoperative treatment strategies.

Our study has a few limitations that should be considered when interpreting the results. The sample size was restricted, and some statistical analyses may be non‐significant due to the limited sample availability. Our study's low event rate for 12a lymph node metastasis (10 events among 89 patients) limited adherence to the 10 events‐per‐predictor rule, potentially leading to overfitting and wider confidence intervals. While we mitigated this by selecting clinically relevant variables, this limitation may affect the generalizability of our findings. Consequently, further studies with larger cohorts are warranted to validate our findings.

## 5 Conclusion

Given the debates surrounding the therapeutic implications of station 12a LN dissection, extensive studies on a larger scale are necessary to delve deeper into potential predictors of station 12a LN metastasis. Our findings suggest that No. 12a lymph node involvement is influenced by metastasis‐related factors and not significantly affected by prior NAC. While these results provide preliminary insights, further studies with robust comparative designs, such as matched analyses or historical case–control cohorts, are needed to confirm whether routine No. 12a lymph node dissection remains necessary in patients treated with NAC.

Although No. 12a lymph node dissection is a technically demanding procedure, it seems that in advanced gastric cancer with preoperative lymph node involvement its dissection should be considered in all cases. However, if preoperative imaging with CT scan or endoscopic ultrasound does not show clear evidence of lymph node involvement, dissection of the No. 12a lymph node may be safely omitted.

## Author Contributions

Amirmohsen Jalaeefar: Conception and design of the study, writing the paper. Habibollah Mahmoodzadeh: Data collection and/or processing, Critical review. Mohammad Shirkhoda: Conception and design of the study, critical review. Ramesh Omranipour: Conception and Design of the study. Seyed Rouhollah Miri: Data collection and/or processing. Narjes Mohammadzadeh: Writing the paper. Arshia Zardoui: Data collection and/or processing, Writing the paper. Amirsina Sharifi: Data collection and/or processing, Writing the paper.

## Ethics Statement

Institutional review board and ethics committee of Tehran University of Medical Sciences approved the study protocol with certification ID of IR.TUMS.IKHC.REC.1401.419.

## Consent

All participants in this study were given information about the study and their role. Therefore, all of them declared their willingness to participate in this study, and informed consent was received.

## Conflicts of Interest

The authors declare no conflicts of interest.

## Supporting information


**Data S1** Supporting Information.

## Data Availability

The data that support the findings of this study are available on request from the corresponding author. The data are not publicly available due to privacy or ethical restrictions.

## References

[1] H. Sung , J. Ferlay , R. L. Siegel , et al., “Global Cancer Statistics 2020: GLOBOCAN Estimates of Incidence and Mortality Worldwide for 36 Cancers in 185 Countries,” CA: A Cancer Journal for Clinicians 71, no. 3 (2021): 209–249, 10.3322/caac.21660. 33538338

[2] W. Dai , E.‐T. Zhai , J. Chen , et al., “Extensive Dissection at No. 12 Station During d2 Lymphadenectomy Improves Survival for Advanced Lower‐Third Gastric Cancer: A Retrospective Study From a Single Center in Southern China,” Frontiers in Oncology 11 (2021): 760963, 10.3389/fonc.2021.760963. 35087750 PMC8787051

[3] Y.‐P. Dong , F.‐L. Cai , Z.‐Z. Wu , et al., “Risk of Station 12a Lymph Node Metastasis in Patients With Lower‐Third Gastric Cancer,” World Journal of Gastrointestinal Surgery 13, no. 11 (2021): 1390–1404, 10.4240/wjgs.v13.i11.1390. 34950428 PMC8649572

[4] P. Shu , X. Sun , F. Liu , et al., “Pattern of No. 12a Lymph Node Metastasis in Gastric Cancer. Chinese,” Journal of Cancer Research 33, no. 1 (2021): 61–68, 10.21147/j.issn.1000-9604.2021.01.07. PMC7941682 33707929

[5] Y.‐F. Zhu , K. Liu , W.‐H. Zhang , et al., “Is No. 12a Lymph Node Dissection Compliance Necessary in Patients Who Undergo D2 Gastrectomy for Gastric Adenocarcinomas? A Population‐Based Retrospective Propensity Score Matching Study,” Cancers 15, no. 3 (2023): 749, 10.3390/cancers15030749. 36765707 PMC9913786

[6] Japanese Gastric Cancer Association jgca@ koto. kpu‐m. ac j , “Japanese Gastric Cancer Treatment Guidelines 2021,” Gastric Cancer 26, no. 1 (2023): 1–25, 10.1007/s10120-022-01331-8. 36342574 PMC9813208

[7] W. J. Seo , C. M. Lee , Y.‐J. Jang , S.‐S. Park , and J.‐H. Kim , “Survival Impact of Compliance in Extra‐Perigastric Lymphadenectomy for Gastric Cancer: 20 Years of Real‐World Data From a Single Institution,” Surgery 171, no. 4 (2022): 948–954, 10.1016/j.surg.2021.09.017. 35094874

[8] N. V. T. Anh , Q. T. Dang , N. L. Vuong , et al., “Regional Lymph Node Metastasis Distribution in Resectable Middle‐Third Gastric Cancer: A Cross‐Sectional Study,” Cureus 15, no. 6 (2023): e41236, 10.7759/cureus.41236. 37397656 PMC10313942

[9] J. A. Ajani , T. A. D'Amico , D. J. Bentrem , et al., “Gastric Cancer, Version 2.2022, NCCN Clinical Practice Guidelines in Oncology,” Journal of the National Comprehensive Cancer Network 20, no. 2 (2022): 167–192, 10.6004/jnccn.2022.0008. 35130500

[10] F. Faul , E. Erdfelder , A. Buchner , and A.‐G. Lang , “Statistical Power Analyses Using G* Power 3.1: Tests for Correlation and Regression Analyses,” Behavior Research Methods 41, no. 4 (2009): 1149–1160, 10.3758/BRM.41.4.1149. 19897823

[11] M. C. Parent , “Handling Item‐Level Missing Data: Simpler is Just as Good,” The Counseling Psychologist 41, no. 4 (2013): 568–600.

[12] S. S. Joshi and B. D. Badgwell , “Current Treatment and Recent Progress in Gastric Cancer,” CA: A Cancer Journal for Clinicians 71, no. 3 (2021): 264–279, 10.3322/caac.21657. 33592120 PMC9927927

[13] F. Lordick , F. Carneiro , S. Cascinu , et al., “Gastric Cancer: ESMO Clinical Practice Guideline for Diagnosis, Treatment and Follow‐Up☆,” Annals of Oncology 33, no. 10 (2022): 1005–1020, 10.1016/j.annonc.2022.07.004. 35914639

[14] T. Nakamura , Y. Hojo , T. Kumamoto , Y. Kurahashi , Y. Ishida , and H. Shinohara , “History of the Lymph Node Numbering System in the Japanese Classification of Gastric Carcinoma Since 1962,” Surgery Today 52, no. 11 (2022): 1–9, 10.1007/s00595-021-02395-2. 34686929

[15] M. B. Amin , S. B. Edge , F. L. Greene , et al., AJCC Cancer Staging Manual (Springer, 2017).

[16] C. Shirong , C. Jianhui , C. Chuangqi , et al., “Survival of Proper Hepatic Artery Lymph Node Metastasis in Patients With Gastric Cancer: Implications for D2 Lymphadenectomy,” PLoS One 10, no. 3 (2015): e0118953, 10.1371/journal.pone.0118953. 25768441 PMC4358929

[17] S. L. Lee , H. H. Lee , Y. H. Ko , et al., “Relevance of Hepatoduodenal Ligament Lymph Nodes in Resectional Surgery for Gastric Cancer,” Journal of British Surgery 101, no. 5 (2014): 518–522, 10.1002/bjs.9438. 24615472

[18] Z.‐L. Li , L.‐Y. Zhao , W.‐H. Zhang , et al., “Clinical Significance of Lower Perigastric Lymph Nodes Dissection in Siewert Type II/III Adenocarcinoma of Esophagogastric Junction: A Retrospective Propensity Score Matched Study,” Langenbeck's Archives of Surgery 407, no. 3 (2022): 985–998, 10.1007/s00423-021-02380-w. 34792614

[19] Y. Woo , B. Goldner , P. Ituarte , et al., “Lymphadenectomy With Optimum of 29 Lymph Nodes Retrieved Associated With Improved Survival in Advanced Gastric Cancer: A 25,000‐Patient International Database Study,” Journal of the American College of Surgeons 224, no. 4 (2017): 546–555, 10.1016/j.jamcollsurg.2016.12.015. 28017807 PMC5606192

[20] Q.‐Y. Chen , Q. Zhong , Z.‐Y. Liu , et al., “Does Noncompliance in Lymph Node Dissection Affect Oncological Efficacy in Gastric Cancer Patients Undergoing Radical Gastrectomy?,” Annals of Surgical Oncology 26 (2019): 1759–1771, 10.1245/s10434-019-07217-x. 30756329

[21] M. D. Boşcaiu , M. Dragomir , B. Trandafir , V. Herlea , and C. Vasilescu , “Should Surgical Ex Vivo Lymphadenectomy Be a Standard Procedure in the Management of Patients With Gastric Cancer? Our Personal Experience, Systematic Literature Review, and Meta‐Analysis,” European Surgery 50 (2018): 169–176, 10.1007/s10353-018-0519-z.

[22] C. de Jongh , L. Triemstra , A. van Der Veen , et al., “Pattern of Lymph Node Metastases in Gastric Cancer: A Side‐Study of the Multicenter LOGICA‐Trial,” Gastric Cancer 25, no. 6 (2022): 1060–1072, 10.1007/s10120-022-01329-2. 36103060 PMC9587950

[23] T.‐H. Kim , I.‐H. Kim , S. J. Kang , et al., “Korean Practice Guidelines for Gastric Cancer 2022: An Evidence‐Based, Multidisciplinary Approach,” Journal of Gastric Cancer 23, no. 1 (2023): 3–106, 10.5230/jgc.2023.23.e11. 36750993 PMC9911619

[24] C.‐D. Zhang , H. Yamashita , Y. Okumura , K. Yagi , S. Aikou , and Y. Seto , “Signature and Prediction of Perigastric Lymph Node Metastasis in Patients With Gastric Cancer and Total Gastrectomy: Is Total Gastrectomy Always Necessary?,” Cancers 14, no. 14 (2022): 3409, 10.3390/cancers14143409. 35884470 PMC9319199

