# Supplementary material for: Clinicopathological Factors Predisposing to No. 12a Lymph Node Metastasis in Gastric Cancer: A Prospective Cohort Analysis
Source: Cancer Rep (Hoboken). 2025 Jun 11;8(6):e70239. doi: 10.1002/cnr2.70239 (PMC12153970; doi:10.1002/cnr2.70239)
Supplement: Supplementary file 1 — Data S1 Supporting Information. [file CNR2-8-e70239-s001.docx]

Supplement:

***Proximal gastrectomy is defined as resection of one third of proximal part of the stomach. Subtotal gastrectomy: Resection of nearly 80% of the stomach.***

***D1 lymphadenectomy:*** ***D1 dissection entails the removal of lymph nodes along the right and left cardiac, lesser and greater curvature, suprapyloric along the right gastric artery, and infrapyloric area.***

***D2 lymphadenectomy:*** ***D2 dissection encompasses all the nodes from D1, in addition to hose along the left gastric artery, common hepatic artery, celiac artery, and splenic artery.***

***LN 12a:*** Station 12a LNs, located along the proper hepatic artery within the hepatoduodenal ligament

***N1: metastasis in 1-2 regional lymph nodes***

***N2: metastasis in 3-6 regional lymph nodes***

***N3: metastasis in 7 or more regional lymph nodes.***

***Perigastric lymph nodes location:***
